# Longitudinal migration and inducible displacement of the Mobility Total Ankle System: Radiostereometry in 23 patients with 2 years of follow-up

**DOI:** 10.3109/17453674.2012.712890

**Published:** 2012-08-25

**Authors:** Michael J Dunbar, Jason W Fong, David A Wilson, Allan W Hennigar, Patricia A Francis, Mark A Glazebrook

**Affiliations:** ^1^Division of Orthopaedics, Department of Surgery; ^2^School of Biomedical Engineering, Dalhousie University, Halifax, Nova Scotia, Canada

## Abstract

**Background and purpose:**

RSA can be used for early detection of unstable implants. We assessed the micromotion of the Mobility Total Ankle System over 2 years, to evaluate the stability of the bone-implant interface using radiostereometric analysis measurements of longitudinal migration and inducible displacement.

**Patients and methods:**

23 patients were implanted with the Mobility system. Median age was 62 (28–75) years and median BMI was 28.8 (26.0–34.5). Supine radiostereometric analysis examinations were done from postoperatively to the 2-year follow-up. Standing examinations were taken from the 3-month to the 2-year follow-up. Migrations and displacements were assessed using model-based RSA software (v. 3.2).

**Results:**

The median maximum total point motion (MTPM) for the implants at 2 years was 1.19 (0.39–1.95) mm for the talar component and 0.90 (0.17–2.28) mm for the spherical tip of the tibial component. The general pattern for all patients was that the slope of the migration curves decreased over time. The main direction of motion for both components was that of subsidence. The median 2-year MTPM inducible displacement for the talar component was 0.49 (0.27–1.15) mm, and it was 0.07 (0.03–0.68) mm for the tibial component tip.

**Interpretation:**

The implants subside into the bone over time and under load. This corresponds to the direction of primary loading during standing or walking. This statistically significant motion may become a clinically significant finding that would correspond with premature implant failure.

Published evidence has supported the use of both ankle arthrodesis (AA) and total ankle arthroplasty (TAA) for the treatment of end-stage arthritis of the ankle (Glazebrook 2010). Current TAA designs have a reported overall 5-year survivorship of approximately 90%. Major complications associated with TAA failure include subsidence, deep infections, and aseptic loosening of components (Glazebrook et al. 2009, Gougoulias et al. 2009, 2010).

A systematic review has indicated that evaluations of TAAs for radiographic loosening have used different methods (Gougoulias et al. 2010). The established value of radiostereometric analysis (RSA) in studying hip replacements (Karrholm et al. 1994) and knee replacements (Ryd et al. 1995) suggests that RSA may be a valuable technique in assessing new designs of TAA.

Validation of new implants and surgical techniques early in the design cycle with high-quality RSA data can screen for inferior technology (Thanner et al. 1995, Nilsson and Dalen 1998, Karrholm et al. 2006). Short-term 2-year RSA results correlate with and predict long-term 10-year clinical results (Karrholm et al. 1994, 2006 , Ryd et al. 1995).

Model-based RSA (MBRSA) avoids the need to attach markers to the implant and instead positions an implant by its radiographic contour (Valstar et al. 2001, Kaptein et al. 2003, 2004, Hurschler et al. 2009, Seehaus et al. 2009). This approach avoids the difficulties of accurately attaching markers to implants, which can be expensive, can be over-projected by the implant itself, and can be detrimental to the implant integrity (Karrholm 1989, Karrholm et al. 2006, Kaptein et al. 2003, 2004).

In longitudinal migration studies, the RSA exams are usually performed under unloaded or supine conditions where each sequential examination compares the implant position with respect to the position of the implant at the postoperative examination. This measure gives the motion of the implant relative to the bone over time. In inducible-displacement studies, the change in position is determined from a loaded or standing examination at a specific point in time and an unloaded or supine examination at the same follow-up occasion. This measure provides the motion of the implant in response to an instantaneous loading.

Like continuous longitudinal migration of a prosthesis, significant inducible displacement of the prosthesis is regarded as a negative finding. The cyclic motion detected by inducible displacement is thought to contribute to clinical loosening, and is thought to reflect the quality of the bone-implant interface (Ryd et al. 1986, Wilson et al. 2009).

The use of MBRSA for assessment of TAA micromotion has been validated in a previous study (Fong et al. 2011). The maximum translation error (MTE), expressed as a standard deviation, was 0.07 mm for the spherical tip of the tibial component and 0.09 mm for the talar component, and the maximum rotational error (MRE) was 0.5° for the talar component (Fong et al. 2011).

We assessed the micromotion of the Mobility Total Ankle System (DePuy, Warsaw, IN) to evaluate the stability of the bone-implant interface using longitudinal migration and inducible displacement measures.

## Patients and methods

### Study group

23 patients underwent TAA using the Mobility implant (Table 1). Patient outcome scores, the Short Form-36 (SF-36), and the ankle osteroarthritis score (AOS) were recorded preoperatively, at 1 year, and at 2 years. One experienced fellowship-trained surgeon (MG) performed all of the surgeries. All the patients had given informed consent. The study was carried out in accordance with the principles of the Helsinki Declaration of 1975, as revised in 2000. The Capital District Health Authority Research Ethics Board approved this study (REB File#: CDHA-RS/2005-051 issued on June 1, 2005).

**Table 1.** Patient demographics^a^ and outcome score. Values are median (range)

Outcome scores	Preoperative	1 year	2 years	MD PO–2yr ^b^	p-value ^c^
SF-36, PCS	26 (17–40)	40 (14–51)	39 (14–59)	10 [20]	0.002
SF-36, MCS	54 (18–69)	52 (33–70)	57 (35–66)	1 [20]	0.7
AOS	58 (33–78)	33 (9–94)	34 (13–78)	–23 [16]	0.0006

**^a^** Sex: 12 M, 11 F; Age: 62 (28–75); and BMI: 28.8 (26.0–34.5)

**^b^** MD PO–2yr is the mean difference between preoperatively and 2 years, where the square brackets show the sample size of the matched pairs

**^c^** Paired 2-tailed t-tests.

### Radiographic set-up

RSA examinations were done using the Halifax Stereo Radiography (SR) Suite (Halifax Biomedical Inc., Halifax, Nova Scotia, Canada). A uniplanar RSA calibration box was used (26 fiducial markers per side and 12 control markers) with 2 radiograph tubes that were each oriented 20° from the vertical. The orientation of the patient with respect to the radiographs was such that the radiographs captured bilateral views of the prosthesis.

### RSA examinations

Uniplanar lateral RSA X-ray examinations were done postoperatively and at the 6-week, 3-month, 6-month, 1-year, and 2-year follow-ups using a supine, unloaded position and were used for the calculation of longitudinal migration. The postoperative RSA exams were performed prior to patients weightbearing. Standing lateral RSA examinations with the body weight equally distributed on both legs were performed at 3 months, 6 months, 1 year, and 2 years, and were used for inducible displacement calculations.

### RSA analysis

CAD models for each of the implant sizes used were provided by the manufacturer. These models were converted to 5,000-element meshes that were used by the model-based RSA software (v. 3.2; Medis Specials, Leiden, the Netherlands).

Longitudinal migration and inducible displacement micromotions were assessed using the same RSA software. Implant micromotions (MTPM, x, y, z, Rx, Ry, Rz) were determined and assessed for each patient using model-based pose estimation (Kaptein et al. 2003, Seehaus et al. 2009), and the implant-based coordinate system (Laende et al. 2009). The x-, y-, and z-axes were aligned to correspond with posterior-anterior, lateral-medial, and inferior-superior or alternatively distal-proximal directions. Subsidence was defined in the inferior direction for the talar component and in the superior direction for the tibial component.

**Figure 1.**
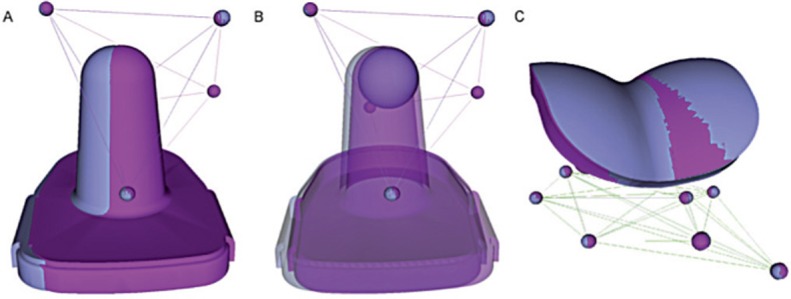
Sample double-examination results for one patient, depicting overlaid implant positions from each RSA examination (gray and magenta), which should be identical. The mismatch in the tibial component (A) showed high imprecision of the system due to the symmetry of the implant. Simplification of the implant to its spherical tip (B) greatly improves the precision. The position of the talar component (C) was precisely repeated in this double examination.

The Elementary Geometric Shapes (EGS) module from the model-based RSA software was used to assess the micromotion of the spherical tip of the tibial component due to implant symmetry (Kaptein et al. 2006) (Figure 1). The issue of implant symmetry with MBRSA has been observed with hip stems (Kaptein et al. 2006, Seehaus et al. 2009). The MTPM of the spherical tip of the tibial implant was the magnitude of the motion of the center of the sphere. Marker models were identified from bone markers and used in cases where there were marker obstructions and/or independent marker migrations (Kaptein et al. 2005).

RSA examinations were removed where the mean error (ME) of rigid body fitting exceeded 0.2 mm because the independent movement of markers within the bone appeared to dominate the migration calculations, resulting in noise within the migration patterns despite being below the suggested 0.35 mm threshold (Valstar et al. 2005). The same threshold has been used previously by this research group for total knee arthroplasty (TKA) (Dunbar et al. 2009).

### Statistics

Custom MATLAB R2010a code (The MathWorks Inc., Natwick, MA) was used to read micromotion in model-based RSA 3.2 output files, to analyze the longitudinal migration and inducible displacement data, to generate plots, and to calculate descriptive statistics. Median (range) was used for MTPM, as it is an unsigned value, and mean (SD) was used for the Cartesian coordinates (x, y, z, Rx, Ry, Rz). Missed follow-up appointments were omitted.

Minitab 16 (Minitab Inc., State College, PA) was used to calculate the demographic, outcome score descriptive statistics, and RSA statistics. Group mean 95% confidence intervals (CIs) were calculated to assess the general micromotion of the implant with respect to the postoperative examination or zero-motion time point. Paired 2-tailed t-tests were used to detect differences between patients at 2 different time points for the SF-36 (PCS and MCS) and AOS values. The significance level for all tests was set at p = 0.05.

## Results

### Outcome scores

The patients as a group showed a statistically significant improvement in outcome scores at 2 years compared to preoperative values, in both PCS (p = 0.002) and AOS (p < 0.0006). The results are summarized in Table 1.

### Longitudinal migration

**Figure 2.**
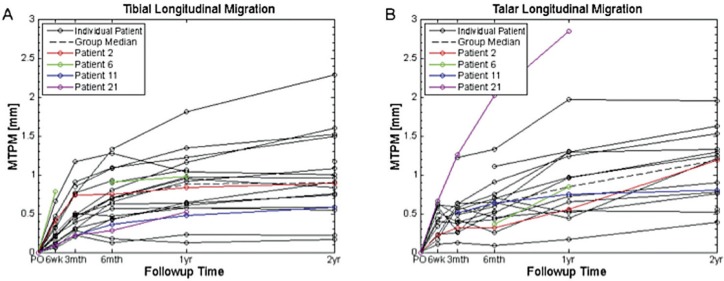
MTPM longitudinal migration of the spherical tip of the tibial component (A) and the talar component (B). Thin black lines: individual patients. Dashed line: group median. Red, green, blue, and magenta: patients who underwent surgical revision. The discontinuities and missed data points indicate where the patients missed follow-up examinations or where the results of the examinations were not usable.

The patient-specific and median longitudinal migration curves in terms of MTPM for the talar components and spherical tips of the tibial components generally showed a high initial rate of MTPM, followed by a reduction in the rate of MTPM over the 2-year follow-up (Figure 2). Some patient-specific migration curves were discontinuous due to missed follow-up appointments, surgical revision, or obscured markers from over-projection. As a result, the median curves contain discontinuous data.

The median (range) MTPM for the implants at the 2-year follow-up were 1.19 (0.39–1.95) mm for the talar implant and 0.90 (0.17–2.28) mm for the spherical tip of the tibial implant (Table 2; missed data points are shown by the reduced sample sizes). Subsidence in the inferior-superior direction was the main direction of movement. This is shown by the 2-year mean CIs that are offset from and do not contain zero. There was high inter-patient variability in all directions, shown by the SDs, which far exceeded the MTE and MRE.

**Table 2.** Longitudinal migration

		Follow-up time			
	3 months	6 months	1 year	2 years	2 year mean 95% CI
Tibial spherical tip, EGS					
n	19	21	20	18	
MTPM (mm)	0.46 (0.21–1.17)	0.70 (0.13–1.33)	0.88 (0.12–1.81)	0.90 (0.17–2.28)	
X: posterior–anterior (mm)	0.06 (0.20)	0.08 (0.28)	0.13 (0.31)	0.16 (0.33)	–0.08 to 0.33
Y: inferior–superior (mm)	0.42 (0.27)	0.56 (0.32)	0.66 (0.36)	0.80 (0.46)	0.58 to 1.03
Z: lateral–medial (mm)	–0.02 (0.23)	–0.04 (0.32)	–0.05 (0.42)	–0.16 (0.44)	–0.38 to 0.06
Tibial component, MBRSA					
n	19	21	20	18	
MTPM (mm)	1.68 (0.28–2.66)	1.67 (0.60–2.71)	1.80 (0.31–3.30)	1.89 (0.41–5.49)	
X: posterior–anterior (mm)	0.04 (0.62)	–0.13 (0.52)	–0.06 (0.71)	–0.18 (0.87)	–0.61 to 0.26
Y: inferior–superior (mm)	0.40 (0.27)	0.56 (0.31)	0.65 (0.38)	0.77 (0.47)	0.54 to 1.01
Z: lateral–medial (mm)	0.11 (0.22)	0.13 (0.26)	0.05 (0.23)	0.03 (0.30)	–0.12 to 0.18
Rx: valgus–varus (°)	–0.4 (1.1)	–0.2 (1.2)	–0.3 (1.3)	–0.4 (1.4)	–1.1 to 0.3
Ry: external–internal rotation (°)	–0.6 (2.6)	–0.9 (2.2)	–0.4 (2.3)	–1.8 (2.8)	–3.2 to –0.4
Rx: dorsiflexion–plantarflexion (°)	0.6 (0.8)	0.6 (1.1)	1.0 (1.2)	0.8 (1.4)	0.1 to 1.5
Talar component, MBRSA					
n	16	18	17	15	
MTPM (mm)	0.50 (0.12–1.26)	0.61 (0.09–2.03)	0.85 (0.17–2.85)	1.19 (0.39–1.95)	
X: posterior–anterior (mm)	0.05 (0.22)	0.08 (0.40)	0.11 (0.39)	0.00 (0.18)	–0.10 to 0.10
Y: inferior–superior (mm)	–0.13 (0.14)	–0.25 (0.24)	–0.43 (0.30)	–0.41 (0.33)	–0.59 to –0.23
Z: lateral–medial (mm)	–0.01 (0.13)	–0.01 (0.16)	–0.01 (0.25)	–0.10 (0.19)	–0.21 to 0.00
Rx: valgus–varus (°)	0.1 (0.5)	0.1 (0.5)	0.5 (0.9)	0.3 (0.9)	–0.2 to 0.8
Ry: external–internal rotation (°)	–0.3 (0.9)	–0.3 (0.7)	–0.4 (1.2)	–0.0 (1.1)	–0.6 to 0.6
Rx: dorsiflexion–plantarflexion (°)	0.5 (0.7)	0.6 (1.3)	0.7 (1.3)	0.2 (1.5)	–0.6 to 1.0

The values are given as mean (SD), except for MTPM which is given as median (range)

Patients 1, 3, 4, 7, and 14 experienced complications during or after surgery, and patients 2, 6, 11, and 21 were surgically revised (Table 3). Patient 19 had the longitudinal migration results omitted due to missed postoperative examinations. Patient 10 was omitted because of substantial independent bone marker migrations (ME > 0.2 mm). Marker models were used for 5 of the 46 talar and tibial rigid bodies.

**Table 3.** Complications and longitudinal migration

			Patient (Follow-up time, year)						
	Pt 1 (2)	Pt 2 (2)	Pt 3 (2)	Pt 4 (2)	Pt 6 (1)	Pt 7 (2)	Pt 11 (2)	Pt 14 (2)	Pt 21 (1)
Tibial spherical tip, EGS									
MTPM (mm)	0.74	0.89	0.55	1.52	0.98	0.17	0.59	0.96	0.53
X: posterior–anterior (mm)	0.12	0.33	0.06	0.14	–0.24	–0.09	–0.19	0.33	–0.27
Y: inferior–superior (mm)	0.65	0.62	0.47	1.48	0.74	0.13	0.38	0.80	0.05
Z: lateral–medial (mm)	–0.34	0.55	–0.27	–0.28	–0.60	–0.07	0.40	–0.43	0.45
Tibial component, MBRSA									
MTPM (mm)	1.98	1.59	1.65	2.28	1.99	0.41	1.39	2.86	2.16
X: posterior–anterior (mm)	1.51	0.19	0.44	–0.15	–1.24	–0.06	–0.26	1.27	1.19
Y: inferior–superior (mm)	0.35	0.57	0.46	1.52	0.76	0.04	0.31	0.68	0.10
Z: lateral–medial (mm)	0.08	0.51	–0.01	–0.32	0.08	0.12	0.11	0.03	0.20
Rx: valgus–varus (°)	–2.4	0.4	–1.0	–0.4	–1.9	0.6	1.3	–1.2	1.1
Ry: external–internal rotation (°)	1.2	–2.9	3.5	–3.1	–2.3	–0.5	–1.7	–3.4	1.5
Rx: dorsiflexion–plantarflexion (°)	0.5	0.6	0.2	1.7	–0.9	0.3	0.8	1.9	2.8
Talar component, MBRSA									
MTPM (mm)	1.53	1.19	0.76	1.95	0.85	n/a	0.80	1.33	2.85
X: posterior–anterior (mm)	0.05	–0.13	–0.08	0.29	–0.02	n/a	–0.34	0.02	1.47
Y: inferior–superior (mm)	–0.76	–0.21	–0.29	–0.76	–0.49	n/a	–0.17	–0.80	–1.05
Z: lateral–medial (mm)	–0.23	0.02	0.11	0.04	0.03	n/a	–0.30	–0.01	0.75
Rx: valgus–varus (°)	–1.1	0.9	0.6	1.9	0.4	n/a	0.9	0.4	3.6
Ry: external–internal rotation (°)	–0.5	–0.1	–0.9	–2.7	1.1	n/a	–0.1	0.7	–2.7
Rx: dorsiflexion–plantarflexion (°)	–2.2	–3.1	–1.3	0.8	0.9	n/a	0.7	1.6	3.3

Patient complications: Pt 1: medial malleolar fracture. Pt 2: implant malaligned, wound healing delayed, superficial infection, packed and treated with antibiotics, scoped and debrided at 3.3 years, revised to fusion at 5 years. Pt 3: heterotopic bone fragment removed. Pt 4: increased ankle pain at 15 months, stress fracture at 17 months. Pt 6: aspirated for pain at 6 months, infection consult at 8 months, arthroscopic removal of loose body at 12 months, revised to Hintegra at 28 months, 2 year RSA exam was not completed. Pt 7: surgical complications (malleolar fracture) and poor outcomes, RSA exams were not usable for talus due to marker overprojection. Pt 11: loose medial body, pain and edema at 3 years, revised to Hintegra at 5.5 years. Pt 14: surgical complications, poor outcomes, pain, debridement, heterotopic bone formation. Pt 21: revised to fusion at 15 months.

Patient 2 was revised to a fusion after 5 years. Patient 2 showed a 3.1° dorsiflexion of the talar component at the 2-year follow-up. This motion fell outside the group mean ± 1.96 SD (Table 2).

Patient 6 had a surgical revision to a Hintegra Total Ankle (Newdeal SA, Lyon, France) after 2 years. RSA examinations were completed only up to 1 year. The tibial component in this patient had an external rotation of 7.6° at the 6-week follow-up before moving back to 2.3° at 1 year. These motions were well beyond the double-examination 95% CI for any sample in the group of 3.4° (Fong et al. 2011); also, the 6-week motion fell outside the group mean ± 1.96 SD. The MTPM for this component was the highest of the group at 6 weeks.

Patient 11 was revised to a Hintegra Total Ankle after 5 years. The talar component had posterior motion of 0.34 mm. This motion did not fall outside the group mean ± 1.96 SD, but it was borderline.

Patient 21 was revised to a fusion after 1 year. There was migration of the talar component outside the group mean ± 1.96 SD for all directions of translation and rotation. The MTPM was 2.85 mm at the 1-year follow-up. The talar component was migrating continuously, and this component had the highest migration in the group (Figure 2).

### Inducible displacement results

Several patients missed follow-up appointments or did not undergo an inducible displacement RSA examination. The median MTPM (range) for the implants at the 2-year follow-up was 0.49 (0.27–1.15) mm for the talar component and 0.07 (0.03–0.68) mm for the spherical tip of the tibial component (Table 4). Subsidence is main direction of movement under load—superior for the tibial component, and inferior for the talar component. This is shown by the 2-year mean CIs, which were offset from and did not contain zero. There was high inter-patient variability in all directions, shown by the SDs, which far exceeded the MTE and MRE.

**Table 4.** Inducible displacement

		Follow-up time			
	3 months	6 months	1 year	2 years	2 year mean 95% CI
Tibial spherical tip, EGS					
n	19	21	22	18	
MTPM (mm)	0.11 (0.03–0.52)	0.10 (0.02–0.43)	0.09 (0.03–0.19)	0.07 (0.03–0.68)	
X: posterior–anterior (mm)	–0.02 (0.08)	–0.03 (0.09)	–0.01 (0.07)	–0.01 (0.33)	–0.09 to 0.08
Y: inferior–superior (mm)	0.05 (0.11)	0.05 (0.04)	0.04 (0.04)	0.02 (0.46)	–0.02 to 0.06
Z: lateral–medial (mm)	–0.04 (0.12)	–0.01 (0.10)	–0.01 (0.06)	–0.01 (0.44)	–0.01 to 0.01
Tibial component, MBRSA					
n	19	21	22	18	
MTPM (mm)	1.34 (0.47–3.50)	1.38 (0.20–5.09)	1.36 (0.20–4.02)	1.34 (0.63–3.68)	
X: posterior–anterior (mm)	–0.22 (0.52)	–0.41 (0.75)	–0.29 (0.70)	–0.35 (0.44)	–0.57 to –0.13
Y: inferior–superior (mm)	0.05 (0.16)	0.09 (0.11)	0.10 (0.11)	0.09 (0.11)	0.04 to 0.15
Z: lateral–medial (mm)	–0.01 (0.25)	–0.07 (0.20)	–0.03 (0.25)	0.01 (0.32)	–0.15 to 0.17
Rx: valgus–varus (°)	0.1 (0.2)	0.1 (0.1)	0.1 (0.1)	0.1 (0.1)	0.0 to 0.1
Ry: external–internal rotation (°)	–0.6 (3.1)	–1.8 (3.4)	–0.8 (3.0)	1.3 (3.5)	–0.4 to 3.0
Rx: dorsiflexion–plantarflexion (°)	–0.8 (1.2)	–0.3 (1.2)	–0.3 (1.0)	–0.6 (0.7)	–1.0 to –0.3
Talar component, MBRSA					
n	18	20	21	18	
MTPM (mm)	0.36 (0.12–1.04)	0.38 (0.07–0.99)	0.40 (0.18–1.30)	0.49 (0.27–1.15)	
X: posterior–anterior (mm)	0.00 (0.10)	0.01 (0.17)	0.04 (0.11)	0.11 (0.10)	0.06 to 0.16
Y: inferior–superior (mm)	–0.04 (0.06)	–0.05 (0.07)	–0.06 (0.08)	–0.06 (0.05)	–0.09 to –0.03
Z: lateral–medial (mm)	–0.02 (0.06)	–0.01 (0.07)	–0.01 (0.12)	0.01 (0.10)	–0.04 to 0.06
Rx: valgus–varus (°)	–0.1 (0.4)	–0.0 (0.4)	0.0 (0.6)	0.0 (0.4)	–0.2 to 0.2
Ry: external–internal rotation (°)	0.1 (0.8)	0.2 (0.5)	0.2 (0.8)	–0.1 (1.2)	–0.7 to 0.5
Rx: dorsiflexion–plantarflexion (°)	–0.1 (0.6)	0.1 (0.8)	0.1 (1.0)	–0.4 (0.5)	–0.6 to –0.1

The values are given as mean (SD), except for MTPM which is given as median (range)

## Discussion

### Longitudinal migration

Thanner et al. (1995) suggested that subsidence may still be the best predictor of painful migration in TAA. Subsidence is the direction of greatest translational motion over time. However, the slope of the subsidence curve appeared to be decreasing and the patient-specific longitudinal subsidence patterns were similar to those seen in a previous RSA publication on TAA (Carlsson et al. 2005).

One publication stated that some complications with TAA are associated with specific TAA implants (Deorio and Easley 2008). 4 surgical revisions were carried out at the time of writing of our article. Patient 2 showed medial tilting of the tibial component and dorsiflexion of the talar component at the 2-year follow-up. The tibial implant of patient 6 migrated more in the initial 6 weeks than any of the other implants. Patient 11 showed posterior motion of the talar component at the 2-year follow-up. Patient 21 required premature revision and showed the greatest migration of the talar component for all follow-up appointments from 6 weeks to 1 year.

This suggests that the mode of failure of this implant is related to failure to obtain early fixation of either of the components. The symmetry of the conical stem may play a role in its failure, as the implant can rotate more freely about the conical axis. This is supported by the fact that the tibial component of patient 6 had an external rotation of 7.6° at 6 weeks. It is unfortunate that the MBRSA pose estimation and related rotations are imprecise, due to the same symmetry that may make this component unstable. This could very well mask the migration of the tibial component in this direction. To permit feedback about the design, these migration data should be examined further as more revision data become available. It is uncertain whether the statistical significance of this motion would match the clinical significance of long-term follow-up.

Continuous migration for TAA may still predict premature failure within 10 years, as seen in TKA (Ryd et al. 1995). Additional research is required to be able to quantify the acceptable early migration of different TAA implant designs and their components. The MTPM threshold of 0.2 mm between the 1-year and 2-year follow-ups used in RSA of TKA may not be suitable for use with TAA. There are anatomical and physiological differences between the ankle and knee joints, which probably factor into this problem. Further studies are required to establish a threshold for continuous migration, such as those identified for the arthroplasty of the other lower extremity joints (Karrholm et al. 1994, Ryd et al. 1995).

### Inducible displacement

Similar to longitudinal migration, but to a lesser degree, group mean inducible displacement was seen as subsidence in the direction of primary loading for the talar and tibial components.

To our knowledge, there has been no literature on inducible displacement in TAA implants. Thus, the inducible displacement results were compared with the published literature on TKA tibial components (Ryd et al. 1986, Uvehammer and Karrholm 2001, Wilson et al. 2009). This established a range of values that could reasonably be expected in this study.

The range of 2-year MTPM inducible displacement for the talar component was 0.27–1.15 mm. This is comparable to that of a TKA tibial component: 0.2–1.0 mm (Ryd et al. 1986). This may be partially accounted for in the elasticity of bones and implants, which is thought to account for up to 0.3 mm of inducible displacement in TKA prostheses (Little et al. 1986, Ryd and Toksvig-Larsen 1993, Wilson et al. 2009). Micromotion in excess of this could represent early pathological micromotion at the bone-implant interface. Long-term follow-up will be required to ascertain the threshold for worrisome inducible displacement in TAA.

The range of 2-year MTPM inducible displacement for the spherical tip of the tibial component was 0.03–0.68 mm. This is comparable to that of knee arthroplasty tibial monoblock components, 0.1–0.4 mm (Wilson et al. 2009). This suggests that the tip of the device may be well constrained within the bone, but it does not rule out rotation of the component, or pivoting of the component about the tip.

The reasons for detecting little or no inducible displacement in terms of MTPM may have been (1) that the tissues around the Mobility were resistant to displacement under these loading conditions, and/or (2) that the measurement sensitivity is too low for this task. Patients guarding their treated leg may tend to support most of their weight with their untreated leg, and therefore contribute to these issues. It was impossible to determine whether this had been occurring with the existing set-up. It is apparent that this is a major drawback of using double leg support as the loaded condition. Control of the loading might have been improved by the use of an apparatus that has been used in other studies of inducible displacement (Ryd et al. 1986, Wilson et al. 2009).

### Clinical implications

We have shown the feasibility of using the precision metrics of MBRSA to assess the micromotion of a TAA prosthesis in widespread clinical studies. Our results neither refute nor support the use of the Mobility TAA conclusively. However, the early failures were detected as outliers using RSA, suggesting that the technique is capable of detecting pathological implant fixation. Only continued follow-up will tell whether other RSA outliers will also fail prematurely. The micromotion patterns as determined by RSA give us insight into possible improvements in implant design.
